# The Multifaceted Role of the Ubiquitin Proteasome System in Pathogenesis and Diseases

**DOI:** 10.3390/biom12070925

**Published:** 2022-07-01

**Authors:** Zane Stekel, Yi Sheng, Wei Zhang

**Affiliations:** 1 Department of Molecular and Cellular Biology, College of Biological Science, University of Guelph, 50 Stone Rd. E, Guelph, ON N1G 2W1, Canada; zstekel@uoguelph.ca; 2 Department of Biology, York University, Room 327B Life Science Building, 4700 Keele Street, Toronto, ON M3J 1P3, Canada; 3 CIFAR Azrieli Global Scholars Program, Canadian Institute for Advanced Research, Toronto, ON M5G 1M1, Canada

**Keywords:** ubiquitin, E3 ligases, deubiquitinases, ubiquitin variants, UCHL1, MDM2, SARS-CoV-2 PLpro, targeted protein degradation, PROTACs, post-translational modification

## Abstract

Ubiquitin is a small protein that is conjugated to target proteins to signal a great number of critical biological processes. Impaired ubiquitin signaling and defects in the ubiquitin proteasome system (UPS) surveillance are implicated in many human diseases, including cancer. Characterization of the physiological roles of UPS components and their regulatory mechanisms is therefore vital for the identification of therapeutic targets and the development of tools and paradigms to better understand and treat human diseases. In this Special Issue, we assembled seven original research and review articles to provide insights on the multifaceted role of the UPS in pathogenesis and disease, covering the areas of molecular and cellular mechanisms of UPS enzymes, biochemical and biophysical characterization strategies, drug development, and targeted protein degradation.

Ubiquitination is a well-conserved protein post-translational modification (PTM), characterized by the covalent attachment of ubiquitin (Ub) or poly-Ub chains to substrate proteins, leading to proteasomal degradation or changes in cellular signaling pathways [1]. This process involves a series of ordered steps, mediated by the enzymatic functions of E1 activating enzymes, E2 conjugating enzymes and E3 ligases (E1→ E2→ E3) [2,3]. Ubiquitination is a dynamic and reversible process in which deubiquitinases (DUBs) can cleave ubiquitin from target proteins [4]. Collectively, all of these functional components form the ubiquitin-proteasome system (UPS), a regulated protein degradation system present in all eukaryotes [5]. The UPS is responsible for the degradation of nuclear, cytosolic, and myofibrillar proteins, therefore critical for the proper maintenance of protein homeostasis in human cells [6]. This Special Issue is dedicated to the new paradigm of ubiquitin biology in diseases and recent developments in therapeutic approaches targeting components of UPS.

Malfunction of the UPS is associated with numerous human diseases including cancer, cardiovascular disease and neurodegenerative disease [5,6]. Particularly, E3 ligases and DUBs are frequently implicated in the progression of these diseases, as they catalyze specific ubiquitination and deubiquitination events that are critical to maintaining normal cellular function. In this issue, Diaz et al. highlighted recent findings regarding the physiological and pathological roles of Cullin-RING E3 ubiquitin ligases (CRLs), which play essential roles in regulating the cardiovascular system through UPS [7]. Further research in this field could lead to the development of therapeutic intervention strategies targeting cardiovascular diseases.

The UPS also plays a critical role in virus-induced cell signaling and immune responses [8,9,10,11]. An emerging area of research has been on SARS-CoV-2 (Severe acute respiratory syndrome coronavirus 2), the virus causing COVID-19. PTMs such as ubiquitination and ISGylation are involved in mediating host-pathogen interactions and antiviral cellular signaling through modulation of key events in innate immunity activation [12]. SARS-CoV-2 encodes a papain-like protease (PLpro) that removes Ub and/or ISG15 (an Ub-like modifier) from human substrate proteins. This allows viral replication and avoidance of host immune system surveillance [12]. An in-depth review in this issue by Vere et al. focused on how human and viral DUBs orchestrate the ubiquitination and ISGylation pathways involved in host-pathogen interactions. These proteins can serve as potential targets for therapeutic development to treat COVID-19 patients.

In the past few years, a combinatorial structure-based protein engineering strategy has been successfully used to develop Ub variants (UbVs) to manipulate UPS components in human cells [3,13,14]. Caba et al. provided a comprehensive overview of DUB pharmacological modulators and tools such as UbVs and activity-based probes (ABPs). These tools can be used to better understand DUBs at the cellular level. Specifically, UbV technology is a promising approach to expanding the library of known DUB inhibitors and can be used as a combinatorial platform for structure-guided drug design [15]. For instance, Hewitt et al. utilized a computational approach to create a UbV-based ABP for a DUB UCHL1 (Ubiquitin C-Terminal Hydrolase L1) as UCHL1 overexpression has been associated with various cancer types and neurodegenerative disorders [16]. UCHL3 is another UCH-family DUB that regulates DNA repair and is overexpressed in many cancers [17,18]. Building on their previous UCHL1 work, Hewitt and colleagues generated a selective triple-mutant UbV-ABP for UCHL3 and validated its function in multiple human cell lines [19].

E3 ligases are involved in many biological activities, as they can control protein stability, function and localization [20]. Targeted inhibition of E3 ligases by small molecules provides opportunities for therapeutic development. MDM2 is an E3 ligase that negatively regulates the tumor suppressor p53 and other proteins involved in DNA repair, cell cycle control and apoptosis pathways. Ilic et al. performed virtual screening against a natural product library, identified, and validated that Hinokiflavone (a biflavonoid) is a potent intracellular inhibitor of MDM2 activity [21]. This study sheds light on developing other E3 ligase inhibitors using natural compounds.

Approximately 20 years ago, researchers devised an attractive strategy to induce targeted protein degradation by artificially recruiting an E3 ligase to a non-native substrate. This study was the first to establish the efficacy of developing proteolysis targeting chimeras (PROTACs) for drug development [22]. Since then, many small-molecule ligands that redirect protein homeostasis machinery were developed for the targeted degradation of disease-related proteins for therapeutics [23]. While there are over 600 E3 ligases encoded by the human genome, the capability of many has yet to be established for this type of application [20,24]. In this issue, Aminu et al. utilized structure-based protein engineering to create what they termed Ubiquitin Variant Induced Proximity (UbVIP). The lab first generated non-inhibitory UbV binders for a selected panel of E3 ligases and screened a library of UbVIPs targeting a DNA damage response protein 53BP1, before identifying two E3 ligases (RFWD3 and NEDD4L) that can be leveraged for targeted protein degradation. This work provides new insights into the identification of novel E3 ligases for PROTACs design [25].

Last but not least, PTMs such as methylation, acetylation and phosphorylation are crucial in regulating various cellular processes [26]. Mass spectrometry-based proteomics studies are the key driving force that led to the detection of the majority of the PTM sites known to date [27,28,29]. In this issue, Lacoursiere and colleagues revealed the Ub and UPS PTM landscape, which was discovered through biochemical, biophysical, and proteomics assays. The potential impacts of these PTMs have on ubiquitin signaling are also discussed, along with implications in human diseases [26].

In conclusion, this Special Issue highlights emerging trends in the UPS field, including new biological functions of UPS components, novel strategies to modulate E3 ligases and DUBs in human diseases, targeted protein degradation, and the functional impacts of UPS PTM events.
